# The modulation of EEG variability between internally- and externally-driven cognitive states varies with maturation and task performance

**DOI:** 10.1371/journal.pone.0181894

**Published:** 2017-07-27

**Authors:** Jessie M. H. Szostakiwskyj, Stephanie E. Willatt, Filomeno Cortese, Andrea B. Protzner

**Affiliations:** 1 Department of Psychology, University of Calgary, Calgary, Alberta, Canada; 2 Hotchkiss Brain Institute, University of Calgary, Calgary, Alberta, Canada; Duke-NUS Graduate Medical School, SINGAPORE

## Abstract

Increasing evidence suggests that brain signal variability is an important measure of brain function reflecting information processing capacity and functional integrity. In this study, we examined how maturation from childhood to adulthood affects the magnitude and spatial extent of state-to-state transitions in brain signal variability, and how this relates to cognitive performance. We looked at variability changes between resting-state and task (a symbol-matching task with three levels of difficulty), and within trial (fixation, post-stimulus, and post-response). We calculated variability with multiscale entropy (MSE), and additionally examined spectral power density (SPD) from electroencephalography (EEG) in children aged 8–14, and in adults aged 18–33. Our results suggest that maturation is characterized by increased local information processing (higher MSE at fine temporal scales) and decreased long-range interactions with other neural populations (lower MSE at coarse temporal scales). Children show MSE changes that are similar in magnitude, but greater in spatial extent when transitioning between internally- and externally-driven brain states. Additionally, we found that in children, greater changes in task difficulty were associated with greater magnitude of modulation in MSE. Our results suggest that the interplay between maturational and state-to-state changes in brain signal variability manifest across different spatial and temporal scales, and influence information processing capacity in the brain.

## Introduction

Recent research suggests that variability in brain signal is an important parameter reflecting information processing capacity and functional integrity [1–3]. Empirical work has shown that brain signal variability tracks changes in cognitive capacity through development from childhood to aged adulthood, and also tracks alterations in cognitive capacity and functional integrity in brain disease (as examples, see [4–7]). In a complex nonlinear system such as the brain, variability facilitates the transition from one network configuration to another, in the presence or absence of external stimulation [8]. A system with too little variability has diminished capacity to explore network configurations, and thus may be restricted to a limited number of states. A system with greater variability, and the capacity for dynamic fluctuations in variability, may efficiently explore multiple network configurations and move amongst many states whenever necessary [9, 10].

The conditions under which variability tracks state-to-state transitions within-subject, and how this might relate to maturation and cognitive performance has been relatively understudied. The few studies that examine state-to-state transitions within-subject suggest that brain signal variability changes dynamically from task to resting-state or to fixation (i.e., viewing a fixation cross during fixation blocks for fMRI) [9, 11], among task conditions with increasing difficulty [9], among task conditions with increasing information associated with a stimulus [12, 13], and in healthy but not in damaged brain tissue [10]. For example, Garret, McIntosh, and Grady [14] examined brain signal variability with fMRI while participants determined whether two faces, presented simultaneously, were the same or different. They manipulated task difficulty by visually degrading some stimuli, and found that brain signal variability was reduced during the more difficult condition. They suggested that the more difficult condition required greater engagement of cognitive resources, leaving fewer resources to prepare for other possible events.

Dynamic fluctuations in variability occur not only with differing task conditions, but also within a trial, at the onset of a stimulus, and post-response. An fMRI study suggests that variability decreases from before to after stimulus onset, and that the magnitude of this decrease correlates with the absolute magnitude of mean signal changes associated with stimulus-related processing [15]. This finding is consistent with computational modeling work which suggests that stabilizing one particular network configuration (i.e., actually settling into one response) reduces brain signal variability and allows for better transmission of response-relevant information [16]. Because stimuli typically are presented in succession, once processing is complete, brain signal variability is expected to return to pre-stimulus levels. To our knowledge, no one has examined this variability shift in humans, but cellular work in macaque monkeys and rats suggest that the return of variability to pre-stimulus levels differs with task conditions, where variability remains lower for a longer period of time when the cell is involved in task processing [17, 18]. Thus, pre-stimulus brain signal variability represents the available repertoire of possible states (i.e., a context in which greater information processing capacity is necessary). Post-stimulus processing stabilizes a given state, thus reducing variability (i.e., a context in which interpretable transmission of information is necessary) and must remain reduced in regions that support task processing while processing is ongoing.

It remains unknown how within-subject state-to-state transitions relate to maturation from childhood to young adulthood, and to cognitive performance. Two recent studies looked at this relationship in the context of old age. Using EEG, Sleimen-Malkoun and colleagues [11] examined how old age affects dynamic variability fluctuations between resting-state and an auditory oddball task. They measured variability using multiscale entropy (MSE), a measure that is sensitive to linear and nonlinear variability, and emphasizes the way signals behave over a range of temporal scales from fine (e.g., over milliseconds) to coarse (over minutes) [19]. They showed that for all adult age groups, variability decreases from resting-state to external task at fine temporal scales and increases from resting-state to external task at coarse temporal scales [11]. They further showed that older adults exhibited more subtle state-to-state modulations in signal variability, both in terms of magnitude of change and spatial extent of change, as compared to younger adults [11]. Using fMRI, Garrett and colleagues [9] demonstrated that older adults exhibit more subtle state-to-state increases in variability from fixation to task at the coarse temporal scales measurable with fMRI, and that these sluggish increases were associated with poorer performance (i.e., slower response times). To our knowledge, there is only one study that examined state-to-state variability modulations during maturation from childhood to adulthood. Misic and colleagues [12] used magnetoencephalography (MEG) to show that brain signal variability was generally greater when viewing upright versus inverted faces. However, they did not directly examine potential differences in the magnitude and spatial extent of state-to-state modulations.

In the current study, we expand on previous work by examining how maturation from childhood to adulthood affects state-to-state modulations in brain signal variability. We measured EEG in children aged 8 to 14 and adults aged 18 to 33 during resting-state, and during a symbol-matching task [20]. This task was simple enough to be suitable for both children and adults, and allowed us to manipulate task difficulty. We first examined changes in brain signal variability between resting-state and task conditions with differing levels of difficulty (high, medium, and low). We then compared changes in brain signal variability between resting-state and within-trial during fixation, after stimulus onset, and after response. Previous studies have shown that in comparison to adults, children exhibited a more diffuse and less distinct default mode network [21, 22], and larger and more deterministic responses to external input [6, 23–26]. We therefore hypothesized that state-to-state modulations in variability would be larger and more widespread in children than in adults.

## Methods

### Ethics statement

Adult participants provided written informed consent, child participants provided assent, and the children’s parents provided written informed consent. The University of Calgary Conjoint Faculties Research Ethics Board approved all procedures, assigning the number REB13-1075.

### Participants

All participants were recruited between March 2014 and May 2015 in Calgary, Alberta, Canada. Twenty-two adults (11 female, mean age = 21.73 years, age range = 18 to 33 years) and 19 children (8 female, mean age = 10.16 years, age range = 8 to 14 years) participated in the study. One child withdrew from the study due to fatigue, and his/her data were not included in the dataset. Therefore, all analyses were conducted with 22 adults and 18 children. Participants had no known neurological or psychological disorders, had not previously suffered a head injury, and were not on any psychotropic medication. All participants were right-handed, with normal or corrected-to-normal vision, and able to perceive colour.

### Symbol-matching task

**Fig 1:**
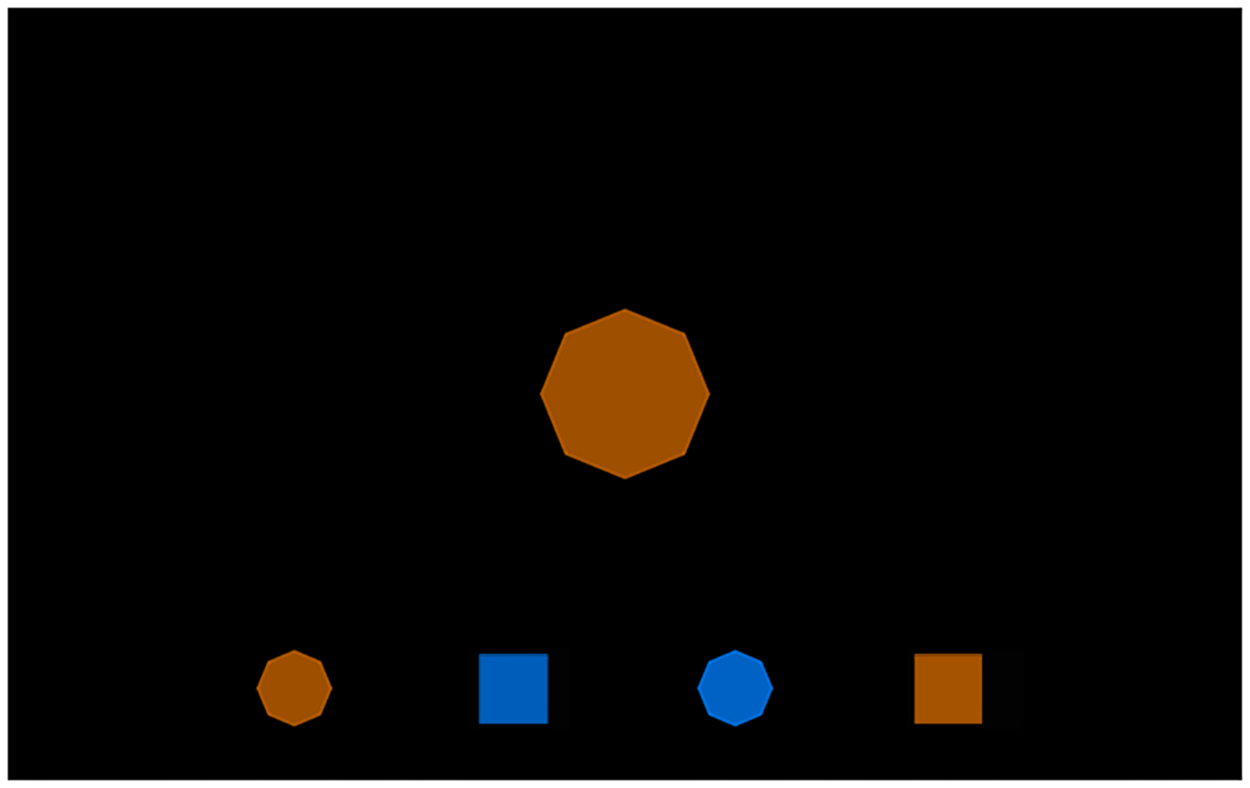
Stimuli example. Example of test stimulus (centre) and reference stimuli (bottom).

Stimuli consisted of coloured geometric shapes, generated in Presentation version 16.1 (Neurobehavioral Systems Inc., Albany, CA, U.S.A.). Stimuli were randomly generated from 15 unique geometric shapes and 24 isoluminant colours (12 pairs of 180° colour compliments, equally spaced around a RGB colour wheel; http://www.workwithcolor.com/hsl-color-picker-01.htm). Each colour was displayed on the testing monitor (HP LP2475w, Hewlett-Packard, Palo Alto, California, USA), and the RGB values were adjusted until luminance values were 15 +/- 5 candelas/m^2^, as measured by a Minolta LS-100 photometer. For each block, two geometric shapes were chosen in two complimentary colours, resulting in four possible stimuli. The *test stimulus* was a large version of one of the four stimuli, and was presented in the centre of the screen for 500ms (Fig 1). All test stimuli were created to have equal area (visual angle = 5 to 9°). The *reference stimuli* were small versions of all four stimuli that were created to have equal length of the longest axis (visual angle = 2°). *Reference stimuli* remained in place for the duration of the block. One test stimulus was presented in each trial, and the participant indicated the position of the reference stimulus that matched the test stimulus by pressing a key (D or F with the left hand, J or K with the right hand) on the keyboard. A jittered inter-trial interval between 1500 and 2000ms followed the response.

### Procedure

We collected eyes open resting-state data prior to task performance. Participants were instructed to relax and look at a fixation cross in the centre of the screen for five minutes. Participants then performed 12 practice trials of the symbol-matching task with stimuli not included in the testing phase. During testing, 18 blocks of 40 trials were presented. We created three difficulty conditions by varying predictability across blocks. The lowest difficulty condition involved one shape being presented considerably more often than the other three (70% and 10% frequency, respectively). The medium difficulty condition involved two shapes being presented more often than the other two (40% and 10% frequency, respectively). The highest difficulty condition contained all shapes appearing with equal proportion (25% frequency). Proportions were randomly assigned to shapes at the beginning of each block. Difficulty conditions appeared in a randomized order.

### Behavioural data

We measured accuracy and response time to assess individual and group performance. We used mixed design Analysis of Variance (ANOVA) to analyse the between-subjects effect of age and the within-subject effect of level of difficulty. Greenhouse-Geisser adjusted values were reported where applicable. Significant interactions were followed up with simple main effects analyses. Significant main effects and simple main effects were followed up with pairwise comparisons. We used Bonferroni correction to maintain a family-wise error rate of 0.05.

### EEG acquisition and preprocessing

Participants were seated in an electrically shielded, soundproof chamber during the experiment. We recorded continuous EEG data from 64 channels with an EasyCap 10/20 positioning system, referenced to Cz, using the BrainVision actiCHamp high-impedance system (Brain Products GmbH, Gilching, Germany). Impedances were maintained at under 17kOhms for the duration of recording. We acquired data at a bandpass of 0.05-100Hz, digitized at a 500Hz sampling rate. After acquisition, we bandpass filtered the continuous data at 0.05-55Hz and re-referenced to the common average reference. We performed independent component analysis (ICA) on the continuous data as implemented in EEGLAB [27]. Components containing artifacts associated with eye movements were removed from the dataset. Four children had excessively noisy channels that required interpolation (maximum of 2 channels per child). Noisy channels were removed from the dataset and interpolated prior to re-referencing so as to not include excessive noise in the common average. ICA analysis was performed excluding the interpolated channels for these children and the interpolated channels were then re-interpolated with the cleaned data. The remainder of preprocessing was consistent for individuals who required interpolation and those who did not.

**Fig 2:**
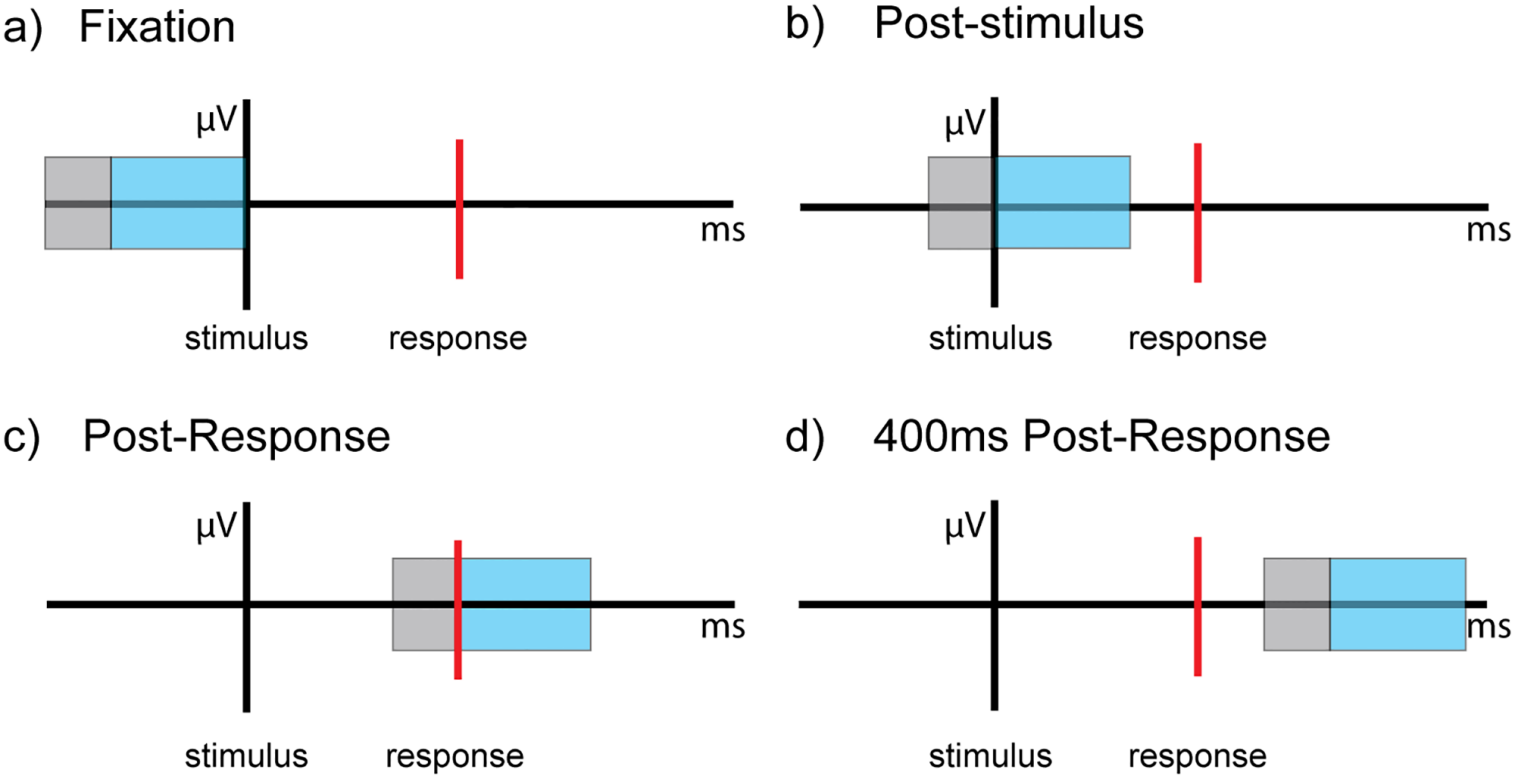
Illustration of epochs over time within a trial. The black vertical line represents stimulus onset, the red vertical line represents response, the blue box represents a 400ms time window, and the grey box represents a 200ms baseline. a) Pre-stimulus epoch, b) post-stimulus epoch, c) post-response epoch, d) 400ms post-response epoch.

For data collected during the symbol-matching task, trials that included stimulus onsets followed by correct responses were binned according to difficulty condition (high, medium, low). Data were segmented differently for task difficulty and within-trial epoch analyses. For the task difficulty analyses, we segmented the data into 1700ms epochs that included 200ms before stimulus onset and 1500ms after stimulus onset. For within-trial epoch analyses, we segmented each trial into four, 600ms epochs: pre-stimulus, post-stimulus, post-response, and 400ms post-response (Fig 2). Each epoch was baseline corrected using the first 200ms portion of the epoch. Data segments used for baseline correction were not included in spectral power density or brain signal variability analyses. Resting-state data were segmented to match the epoch length from the task for within-trial and task difficulty analyses.

### Brain signal variability analysis

Brain signal variability was quantified using MSE, a measure that is sensitive to linear and nonlinear variability [19, 28]. We calculated MSE in two steps, with the algorithm available at www.physionet.org/physiotools/mse/. First, the algorithm progressively down samples EEG data for each trial at each electrode by averaging data points within non-overlapping windows of time. Specifically, for a given temporal scale *t*, the algorithm averages data points within non-overlapping windows of length *t*, and repeats this procedure for increasing values of *t* (e.g., temporal scale t = 1 is the original raw time series, temporal scale t = 2 averages over 2 time points, etc.). Next, the algorithm calculates sample entropy for each temporal scale, measuring predictability in the signal by evaluating the appearance of repetitive patterns. We used a pattern length of *m* = 2, which means we were looking for repeating patterns that are 2 consecutive points in length. We used a similarity criterion of *r* = 0.5 which means two points are considered similar if the difference in amplitude is less than or equal to 50% of the standard deviation for the time course. For each subject, electrode and condition specific MSE estimates were obtained as a mean of the single trial entropy measure for each temporal scale. We required a minimum of 50 time points to reliably estimate sample entropy for a given temporal scale, consistent with previous work [5, 6, 10]. For 600ms epochs (the last 400ms of which was used for analyses), there were 200 data points with a 500Hz sampling rate, allowing us to reliably estimate sample entropy for up to 8ms temporal scales. For 1700ms epochs (the last 1500ms of which was used for analyses), there were 750 data points with a 500Hz sampling rate, allowing us to reliably estimate sample entropy for up to 30ms temporal scales.

### Spectral power density

In addition to MSE, we calculated spectral power density (SPD). A comparison of MSE and SPD results allowed us to examine the relationship between MSE measures and the frequency content of the EEG signal, and to evaluate if maturation-related changes consist of more linear (assessed by both MSE and SPD) or nonlinear (assessed only by MSE) dependencies in the data [7, 29]. A linear model predicts a proportional relationship between causes and effects, whereas this relationship in a nonlinear system is not as straightforward. The combination of analyses therefore provides additional information about brain dynamics that cannot be captured with either type of analysis alone [30].

SPD was calculated using fast Fourier transform of single trial data using in-house code. The signal was normalized to account for any global power change between individuals and relative contributions of different frequency bands to the total spectral power were calculated based on the normalized data. Given a sampling rate of 500Hz and 750 time points in a 1500ms epoch for the difficulty analysis and 200 time points in 400ms within-trial epochs, the frequency resolution was 0.67 and 2.5Hz, respectively.

### Partial least squares snalysis

MSE and SPD measures were statistically assessed with partial least squares (PLS) analysis [6, 31, 32], a multivariate approach that allowed us to identify large-scale group- and condition-dependent changes in the spatiotemporal distributions of MSE and SPD measures. In brief, PLS extracts latent variables (LVs) that identify patterns of similarities or differences in a brain signal measure between conditions and groups. In the most common usage of PLS, contrasts across groups or conditions are not specified in advance. Rather, the algorithm extracts LVs explaining the covariance between groups/conditions and brain signal measure in order of the amount of covariance explained with the most explained listed first. We, instead, used a *nonrotated* version of task-PLS to allow us to test a priori hypotheses with pre-specified contrasts, which are provided for each analysis below. Both versions of task-PLS begin by creating a data matrix with subjects and conditions as rows, and MSE values at all temporal scales/SPD at all frequencies for every electrode as columns. PLS uses singular value decomposition to extract LVs, which contain three vectors. The first vector consists of a singular value, which indicates the strength of the effect expressed by the LV. The remaining two vectors relate experimental design and the brain signal measure. The experimental design vector contains design saliences, which indicate the degree to which each condition within each group is related to the MSE/SPD pattern identified in the LV. These design saliences can be interpreted as the contrast that codes the effect depicted in the LV. The brain signal vector contains MSE/SPD saliences. These are numerical electrode weights that identify the collection of electrodes and temporal scales or frequencies that, as a whole, are most related to the effects expressed in the LV. For each LV, there is one salience per electrode temporal scale or frequency that applies for all groups and all conditions. To obtain summary measures of each participant’s expression of an LV, we calculated brain scores by multiplying the vector of electrode weights by the observed value of the brain signal measure and summing over all brain signal measures for each participant. These brain scores were calculated for each condition and then mean centred using the grand mean across all conditions.

Statistical assessment in PLS is done across two levels. First, the overall significance of each LV is assessed with permutation testing [33]. For each subject, sampling without replacement is used to reassign the order of conditions. PLS is calculated for each sample and the number of times a singular value exceeds the observed singular value relative to the total number of permuted samples is used to assess significance. An LV was considered significant if the observed singular value exceeded the permuted singular value in more than 95% of the permutations (*p* < 0.05). Second, bootstrap resampling is used to estimate confidence intervals around electrode weights in each LV. Bootstrap samples were created by re-sampling subjects with replacement and PLS was recalculated for each new sample. Distributions of bootstrapped values were used to create standard errors for electrode weights and confidence intervals for averaged brain scores, allowing for an assessment of the relative contribution of particular electrodes and temporal scales/frequencies, and the stability of the relationship with age group [34, 35]. No corrections for multiple comparisons are necessary because the electrode saliences are calculated in a single mathematical step on the whole brain. For this paper, we arbitrarily designated a bootstrap ratio threshold of 3.10, corresponding approximately to a 99.9% confidence interval, or a *p* value < 0.001 to display our effects.

We followed-up significant group by task interactions identified through PLS by testing the simple main effect of task in children and adults separately, using task-PLS without pre-specified contrasts. In addition, we conducted pairwise contrasts on the brain scores obtained from separate group analyses to confirm differences among task conditions, and calculated the effect size parameter Cohen’s *d* [36] to compare the magnitude of the task effect in children and in adults. We used a pooled standard deviation estimate from the brain scores that was not corrected for repeated measures to calculate Cohen’s *d* to ensure the effect sizes were not inflated, and more comparable across analyses [37]. We used the Bonferroni adjustment to maintain a family-wise error rate of 0.05, treating each age group as a family.

## Results

### Behavioural results for the symbol-matching task

**Fig 3:**
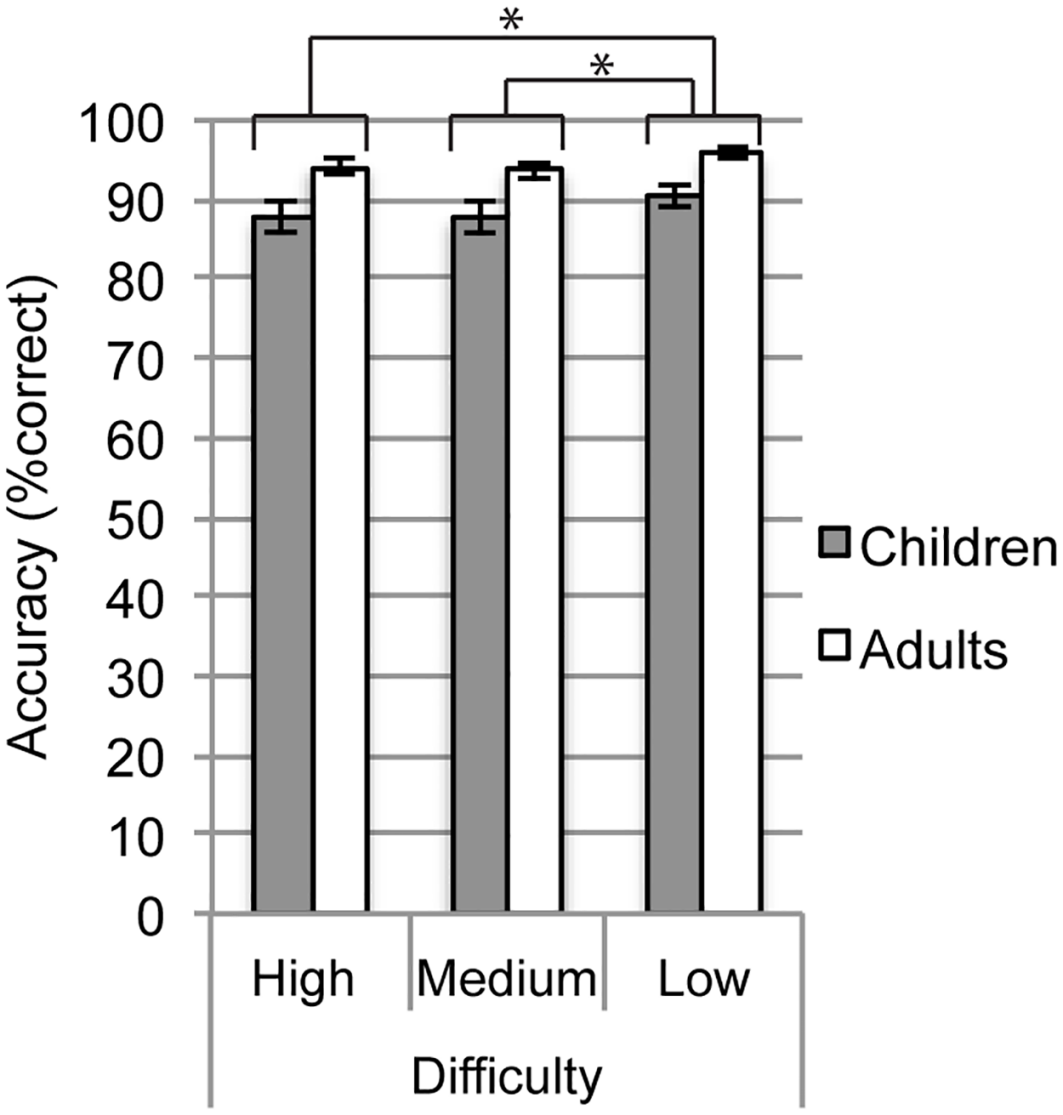
Accuracy in children and adults. Error bars represent standard error. * indicates a significant pairwise comparison at α = 0.017. Pairwise comparisons for accuracy were conducted at the main effect level with children and adults together due to a lack of significant interaction between age group and difficulty conditions.

We used a 2 x 3 omnibus mixed design Analysis of Variance (ANOVA) to examine whether accuracy differed across group (children and adults) and difficulty condition (high, medium, and low) (Table 1) during the symbol-matching task. There was a significant main effect of group indicating that adults (*M* = 94.51, *SD* = 4.22) were more accurate than children (*M* = 88.66, *SD* = 7.54). The main effect of difficulty condition also was significant, and the interaction between group and difficulty condition was not significant. Therefore, we conducted pairwise comparisons among difficulty conditions with children and adults together using an α value of 0.05/3 (0.017) to maintain family-wise error at 0.05 (Table 2). Children and adults were significantly more accurate for the low difficulty condition compared to the high difficulty condition, and the low difficulty condition compared to the medium difficulty condition. Accuracies for high and medium difficulty were not significantly different. These results are illustrated in Fig 3.

**Table 1:** Mixed design ANOVA results for behavioural data.

DV	Effect	df	*F*	*p*	Partial η^2^
**Accuracy**	Age	(1, 38)	11.377	0.002	0.23
	Difficulty	(1.835, 69.722)	6.665	0.003	0.149
	Age*Difficulty	(1.835, 69.722)	0.294	0.727	0.008
**RT**	Age	(1, 38)	34.215	<0.001	0.474
	Difficulty	(1.963, 74.606)	77.628	<0.001	0.671
	Age*Difficulty	(1.963, 74.606)	7.143	0.002	0.158

**Table 2:** Follow-up tests for behavioural data.

DV	Age group	Difficulty	df	*F*	*p*	Partial η^2^
**Accuracy**	All	High—Low	(1, 38)	9.098	0.005	0.193
		High—Medium	(1, 38)	0.181	0.673	0.005
		Medium—Low	(1, 38)	8.693	0.005	0.186
**RT**	Children	High—Low	(1, 17)	76.32	<0.001	0.818
		High—Medium	(1, 17)	5.95	0.026	0.259
		Medium—Low	(1, 17)	38.08	<0.001	0.691
	Adults	High—Low	(1, 17)	50.651	<0.001	0.707
		High—Medium	(1, 17)	13.89	0.001	0.398
		Medium—Low	(1, 17)	34.03	<0.001	0.618

**Fig 4:**
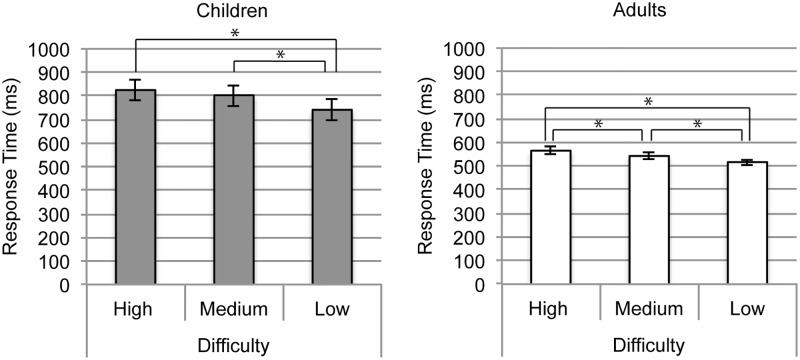
Response time in (a) children and (b) adults. Error bars represent standard error. * indicates a significant pairwise comparison at α = 0.017. Pairwise comparisons of difficulty conditions were conducted for children and adults separately due to a significant interaction between age group and difficulty level.

We used a 2 x 3 omnibus mixed design ANOVA to examine whether mean response time differed over group (children and adults) and difficulty condition (high, medium, and low) (Table 1). The main effect of group was significant, such that adults had faster response times (*M* = 541.9, *SD* = 63.49) than children (*M* = 789.9, *SD* = 188.8). The main effect of difficulty condition was significant, as well as the interaction between age group and difficulty condition. Therefore, we conducted pairwise comparisons of difficulty conditions for children and adults separately, using an α value of 0.05/6 (0.008) to maintain family-wise error at 0.05 (Table 2). For children, response times were significantly longer for high difficulty than low difficulty conditions, and medium difficulty than low difficulty conditions. Children’s response times for high and medium difficulty conditions were not significantly different with the adjusted α value (0.008). For adults, response times were significantly longer for high difficulty than low difficulty conditions, low difficulty than medium difficulty conditions, and medium difficulty than low difficulty conditions. These results are summarized in Fig 4.

To better understand the nature of the interaction, we conducted linear contrasts comparing difference in response time between high and low difficulty, high and medium difficulty, and medium and low difficulty, between children and adults. We used an α value of 0.05/3 (0.017) to maintain family-wise error at 0.05. We found that the difference between high and low difficulty conditions was significantly greater in children (*mean difference* = 87.79, *SD* = 40.68), than in adults (*mean difference* = 48.56, *SD* = 32.01), *F*(1, 38) = 9.395, *p* = 0.004, *η*^*2*^ *=* 0.198, and the difference between medium and low difficulty conditions was significantly greater in children (*mean difference* = 61.49, *SD* = 42.27) than in adults (*mean difference* = 25.98, *SD* = 20.89), *F*(1, 38) = 11.95, *p* = 0.001, *η*^*2*^ *=* 0.240. The difference between high and medium difficulty was not significantly different between children and adults, *F*(1, 38) = 0.851, *p* = 0.978. *η*^*2*^ *<*0.001.

### Neuroimaging results comparing resting-state and the symbol-matching task

#### MSE

**Fig 5:**
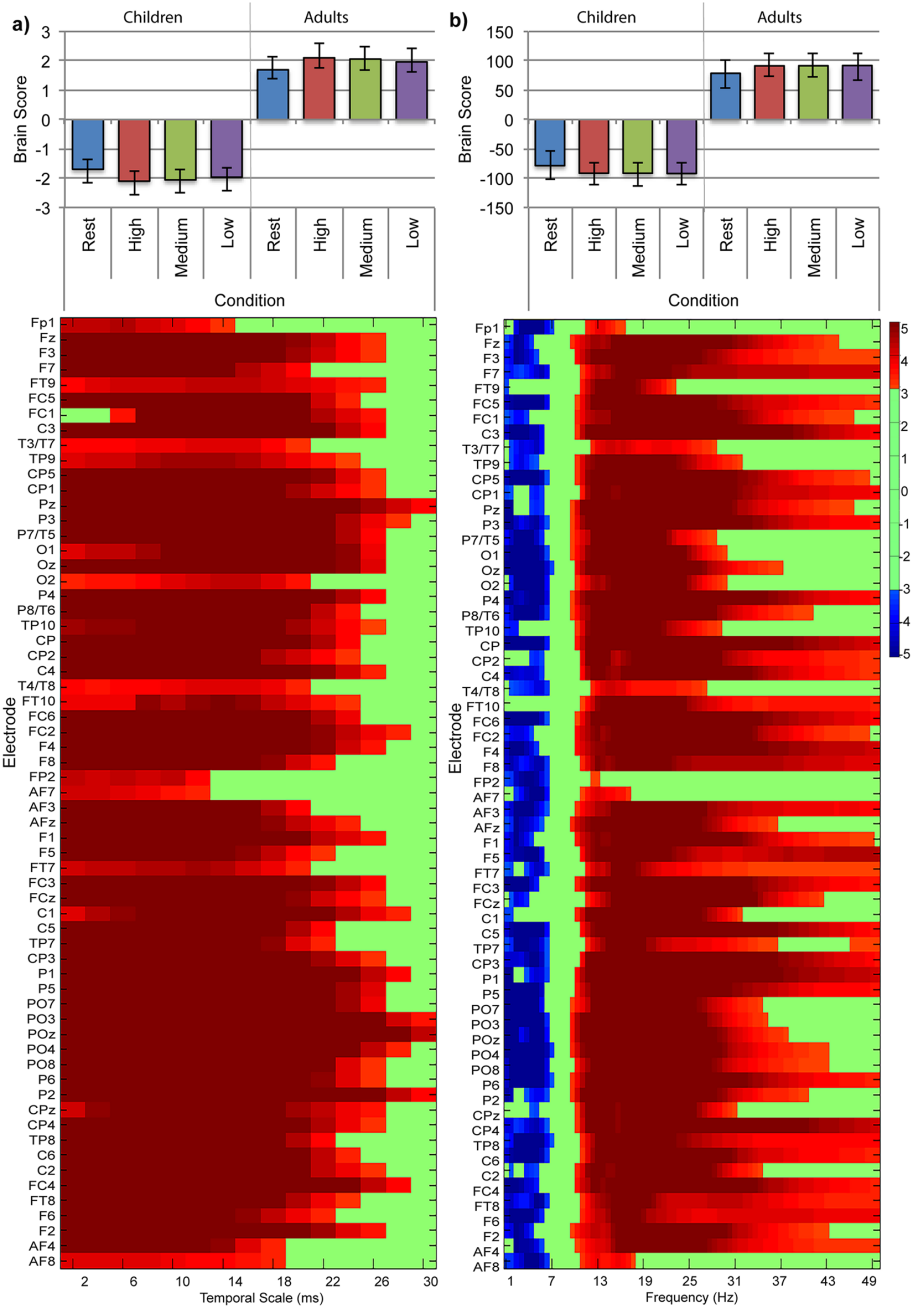
Nonrotated task-PLS of (a) MSE data and (b) SPD data for the two-group resting-state and symbol-matching task analysis: LV1, main effect of age group (children and adults) with resting-state (rest) and symbol-matching conditions (high, medium, and low difficulty). The bar graph (top) shows averaged brain scores for the main effect of age with (a) MSE and (b) SPD, with 95% confidence intervals derived from bootstrap estimation. The statistical plots (bottom) show the main effect of age bootstrap ratios for each electrode and temporal scale. Positive bootstrap ratios (> 3.10) indicate regions that demonstrate the relationship between conditions as displayed in the bar graph (i.e., lesser (a) MSE and (b) SPD for children than adults). Negative bootstrap ratios (< -3.10) indicate regions that demonstrate the opposite relationship between conditions as displayed in the bar graph (i.e., greater (b) SPD for children than adults).

**Fig 6:**
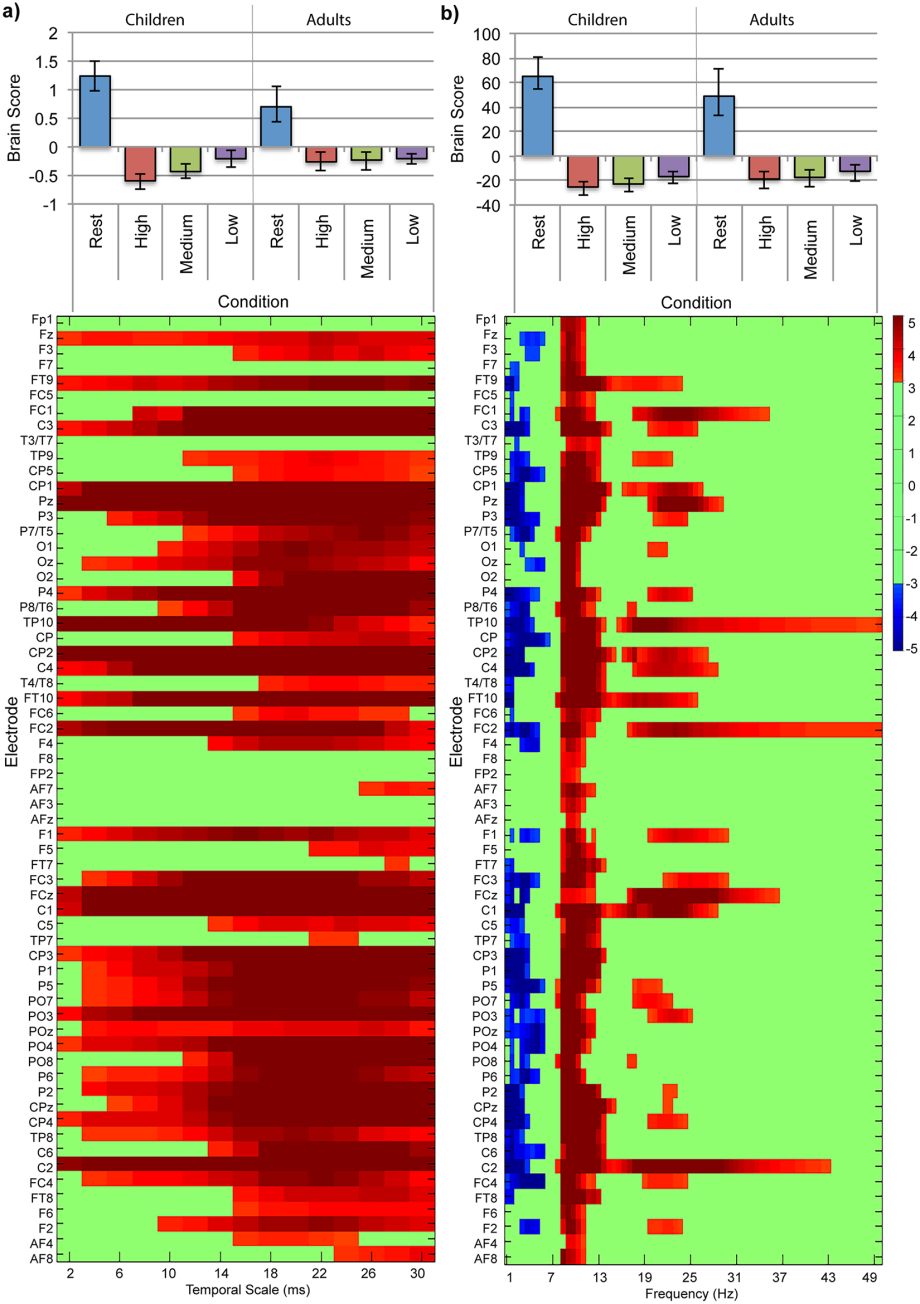
Nonrotated task-PLS of (a) MSE data and (b) SPD data for the two-group resting-state and symbol-matching task analysis: LV2, main effect of task (rest, and high, medium, low difficulty) in children and adults. The bar graph shows averaged brain scores (top) for the main effect of task with (a) MSE and (b) SPD, with 95% confidence intervals derived from bootstrap estimation. The statistical plots (bottom) show the main effect of age bootstrap ratios for each electrode and temporal scale. Positive bootstrap ratios (> 3.10) indicate regions that demonstrate the relationship between conditions as displayed in the bar graph (i.e., greater (a) MSE and (b) SPD for resting-state; Rest, than for task conditions; High, Medium, Low). Negative bootstrap ratios (< -3.10) indicate regions that demonstrate the opposite relationship between conditions as displayed in the bar graph (i.e., lower (b) SPD for Rest, than for task conditions; High, Medium, Low).

We used nonrotated task-PLS of MSE values to test for a main effect of group (children vs. adults, contrast weights: 1, 1, 1, 1, -1, -1, -1, -1), a main effect of task (resting-state vs. high, medium, and low difficulty conditions, contrast weights: 3, -1, -1, -1, 3, -1, -1, -1), and an interaction between these effects (contrast weights: 3, -1, -1, -1, -3, 1, 1, 1). The main effect of group was significant, such that adults had greater MSE than children in resting-state and across all difficulty conditions (*p* < 0.001, Fig 5a). This effect was reliably expressed across all temporal scales tested, but strongest at scales less than 25ms for all electrode sites. The main effect of task was significant, such that brain signal variability was greater during resting-state than during the symbol-matching task (*p* = 0.006, Fig 6a). Additionally, confidence intervals did not overlap for high and low difficulty in children, suggesting that MSE was lower for high difficulty conditions than low difficulty conditions in children. This pattern was reliably expressed for primarily coarse temporal scales (greater than 10ms) across electrode sites in all regions. As the interaction between age group and task was significant (*p* = 0.006), we explored the effect of task in children and adults separately.

**Fig 7:**
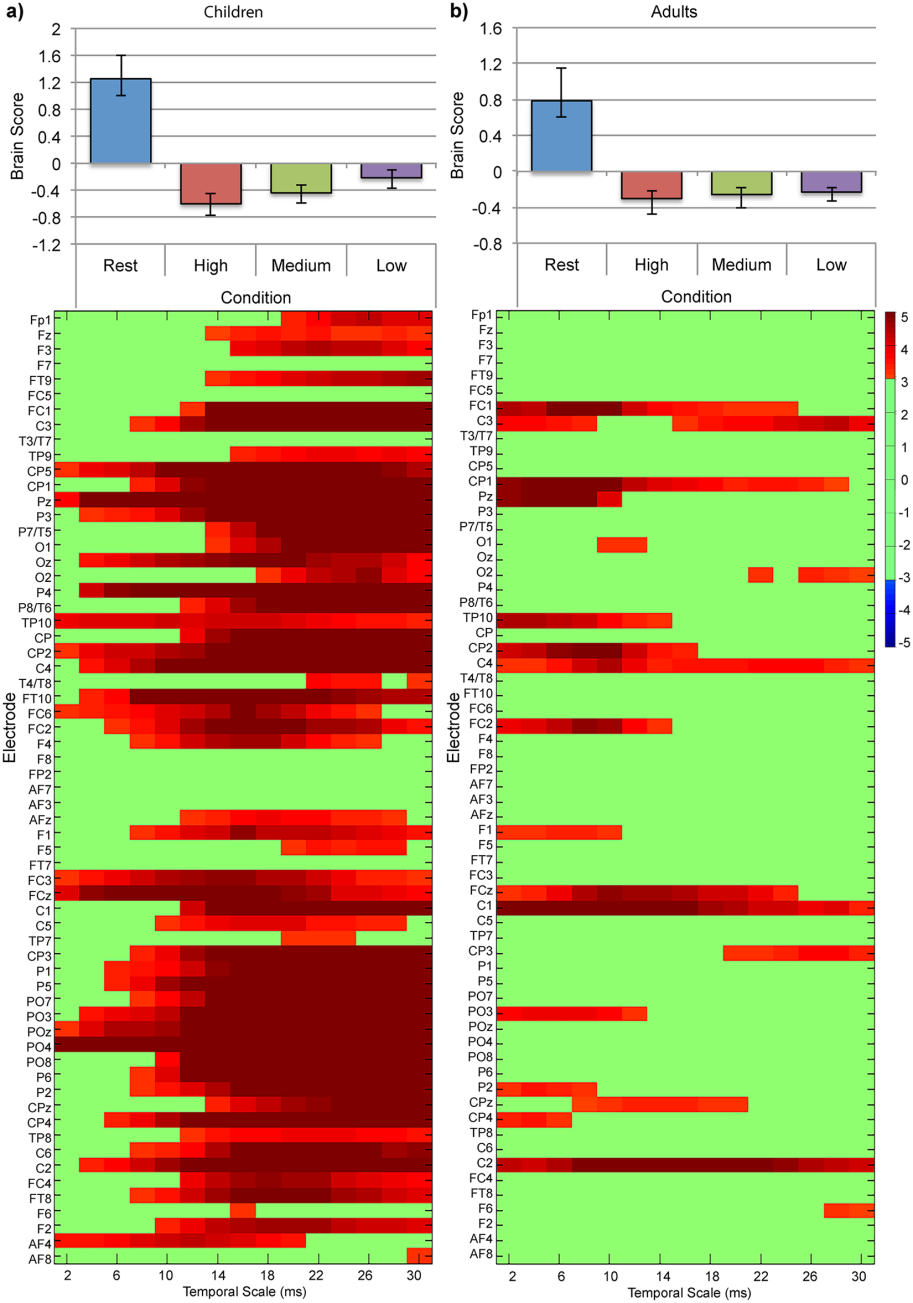
Task-PLS of MSE data for the resting-state and symbol-matching effect in (a) children and (b) adults. The bar graphs (top) show averaged brain scores for LV1 for (a) children and (b) adults with 95% confidence intervals derived from bootstrap estimation. The statistical plots (bottom) show LV1 bootstrap ratios for each electrode and temporal scale. Positive bootstrap ratios (> 3.10) indicate regions that demonstrate the relationship between conditions as displayed in the bar graph (i.e., greater MSE values for resting-state; Rest, than for task conditions; High, Medium, Low).

A task-PLS analysis of MSE values in children revealed one significant LV that differentiated resting-state from the symbol-matching task such that MSE values were greater for resting-state than the three difficulty conditions (*p* = 0.002, Fig 7a). Additionally, confidence intervals for the high and low difficulty conditions did not overlap, suggesting that there were reliable differences in MSE among difficulty conditions. This pattern was reliably expressed for all temporal scales across frontal, central, temporal, and parietal electrode sites, and for coarser temporal scales (scales greater than 15ms) for electrode sites throughout the scalp. A task-PLS analysis of MSE values in adults revealed one significant LV that differentiated resting-state from the symbol-matching task, but did not differentiate difficulty conditions from each other (*p <* 0.001, Fig 7b). This pattern was reliably expressed for most temporal scales across primarily central electrode sites. Further examination using a more lenient threshold of 0.05 (bootstrap ratios > |2.00|) found more frontal, central, and parietal electrodes were reliable for fine temporal scales (less than 10ms), and several more frontal electrodes were reliable for coarser temporal scales (greater than 15ms). These regions became similarly reliable in both groups, resulting in the same overall impression that the effect was more widespread in children than adults and suggesting the difference in spatiotemporal distribution was not due to our chosen thresholding value (data not shown). Thus, our findings suggest that state-to-state modulations in MSE are more widespread in children than in adults, and may also differ in magnitude.

To directly examine the potential group difference in the magnitude of MSE changes, we conducted pairwise contrasts on the brain scores from separate group analyses. We used an α value of 0.0042 to maintain family-wise error at 0.05 (0.05/12 = 0.0042). To examine whether there was a group difference in the magnitude of the change from resting-state to task, we contrasted resting-state to the average of the three difficulty conditions and calculated effect size. We found that for children, dynamic changes in MSE differentiated resting-state (*M =* 1.254, *SD* = 1.798) from the average of the three difficulty conditions (*M* = -0.4179, *SD* = 2.185), *t*(17) = 6.634, *p* < 0.001, *d* = 0.835, and similarly for adults, dynamic changes in MSE differentiated resting-state (*M =* 0.7858, *SD* = 1.401) from the average of the three difficulty conditions (*M* = -0.2619, *SD* = 1.179), *t*(21) = 4.065, *p* < 0.001, *d* = 0.809. To examine whether there were differences in dynamic changes in MSE from state-to-state within the task, we contrasted each of the difficulty conditions. We found that in children, dynamic changes in MSE differentiated the low difficulty condition (*M* = -0.2146, *SD* = 2.133) from the high difficulty condition (*M =* 0.6009, *SD* = 2.200), *t*(18) = -3.198, *p* = 0.003, *d* = 0.1034. No other comparison amongst difficulty conditions was significant for children or adults.

As reported above, our behavioural analyses suggest that the difference in response time among difficulty conditions (i.e., between high and low difficulty, and between high and medium difficulty) was significantly larger in children than adults. To test whether greater dynamic changes in MSE between difficulty conditions in children was due to greater success of the difficulty manipulation for children, we used a residualisation procedure [38, 39] to remove the between-subjects variation related to response time (RT) and accuracy from the MSE data, and repeated our PLS analyses. In both cases, this residualisation removed the difficulty condition differentiation for children, but did not influence the spatial extent of the effect (i.e., MSE effects remained more widespread in children as compared to adults; RT, LV1, *p* < 0.001; accuracy, LV1, *p* < 0.001). To further confirm the lack of MSE differentiation between difficulty conditions, we compared brain scores for resting-state and task conditions from the residualised analyses using t-tests and found significant differences only between resting-state and the average of the three difficulty conditions in each group. For brain scores residualised with RT in children, resting-state (*M* = 1.254, *SD =* 1.860) was higher than the average of all difficulty conditions (*M* = -0.4179, *SD =* 2.418), *t*(17) = 6.671, *p* < 0.001. *d* = 0.775; in adults, resting-state (*M* = 0.7851, *SD =* 1.546) was higher than the average of all difficulty conditions (*M* = -0.2617, *SD =* 1.077), *t*(21) = 3.989, *p* = 0.001, *d* = 0.786. For brain scores residualised with accuracy in children, resting-state (*M* = 1.254, *SD =* 1.551) was higher than the average of all difficulty conditions (*M* = -0.4179, *SD =* 1.919), *t*(17) = 6.661, *p* < 0.001, *d* = 0.958; in adults, resting-state (*M* = 0.7846, *SD =* 1.187) was higher than the average of all difficulty conditions (*M* = -0.2615 *SD =* 1.339), *t*(21) = 3.962, *p* = 0.001, *d* = 0.823.

#### SPD

We used nonrotated task-PLS on spectral power to test for a main effect of group (children vs. adults, contrast weights: 1, 1, 1, 1, -1, -1, -1, -1), a main effect of task (resting-state vs. high, medium, and low difficulty conditions, contrast weights: 3, -1, -1, -1, 3, -1, -1, -1), and an interaction between these effects (contrast weights: 3, -1, -1, -1, -3, 1, 1, 1). The main effect of group was significant such that children had greater SPD at lower frequencies, and lesser SPD at higher frequencies (*p* < 0.001, Fig 5b). Greater SPD for children in the delta band (0.5-3Hz) and theta band (4—7Hz) was reliably expressed across all electrode sites except FT9 and FT10. Lesser SPD for children in the alpha (8—12Hz), beta (13—40Hz), and low gamma (40—50Hz) bands was reliably expressed across all electrode sites except Fp2. The main effect of task was significant such that resting-state differed from all difficulty conditions in the symbol-matching task (*p* < 0.001, Fig 6). More specifically, SPD in the delta (0.5—3Hz) and theta (3—7Hz) bands was lower during resting-state than during the symbol-matching task across primarily central, parietal, temporal, and occipital electrode sites. SPD in the alpha (8—12Hz) band was greater during resting-state than during symbol-matching task for all electrode sites, and SPD for beta (13—40Hz), and low gamma (40—50Hz) bands was greater during resting-state than during the symbol-matching task primarily at central electrode sites. As the interaction between group and task was significant (*p* = 0.015), we explored the effect of task in children and adults separately.

**Fig 8:**
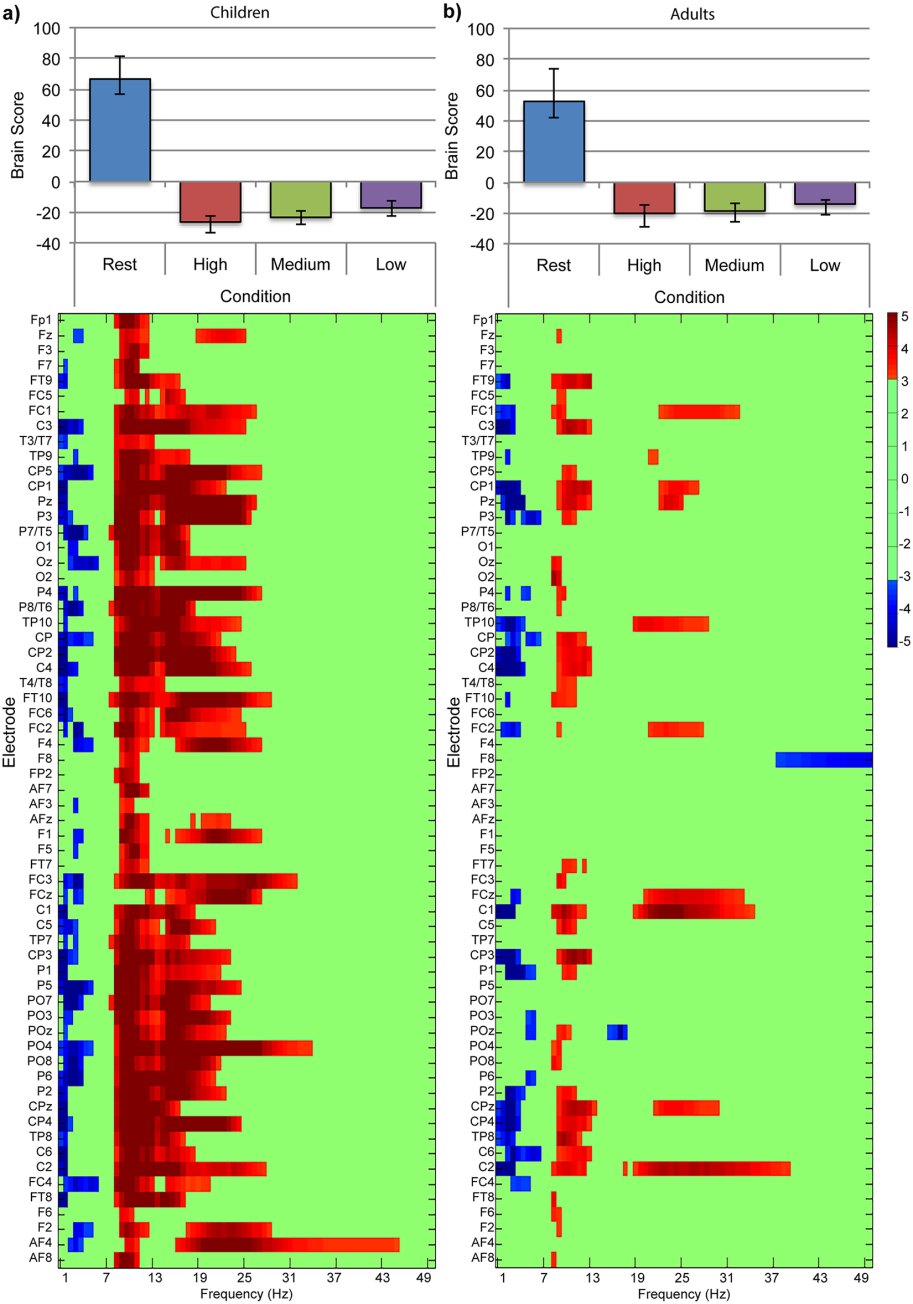
Task-PLS of SPD data for the resting-state and symbol-matching task effect in (a) children and (b) adults. The bar graphs (top) show averaged brain scores for LV1 for (a) children and (b) adults with 95% confidence intervals derived from bootstrap estimation. The statistical plots (bottom) show LV1 bootstrap ratios for each electrode and temporal scale. Positive bootstrap ratios (> 3.10) indicate regions that demonstrate the relationship between conditions as displayed in the bar graph (i.e., greater MSE values for resting-state; Rest than for task conditions; High, Medium, and Low). Negative bootstrap ratios (< -3.10) indicate regions that demonstrate the opposite relationship between conditions as displayed in the bar graph (i.e., lower SPD for Rest, than for task conditions; High, Medium, Low).

A task-PLS analysis of SPD in children revealed one significant LV differentiating resting-state from all difficulty conditions (*p* < 0.001, Fig 8a). SPD was reliably lower in delta (0.5—3Hz) and theta (4—7Hz) bands during resting-state than during the symbol-matching task for most electrode sites across all regions. SPD was reliably greater in the low alpha (8—12Hz) band during resting-state than during the symbol-matching task in all electrode sites, and in the beta (13—40Hz), and low gamma (40—50Hz) bands for most central, parietal, temporal, and occipital electrode sites. A task-PLS analysis of SPD in adults revealed one significant LV differentiating resting-state from all difficulty conditions (*p* < 0.001, Fig 8b). SPD was reliably lower in delta (0.5—3Hz) and theta (4—7Hz) bands during resting-state than during the symbol-matching task, and reliably greater during resting-state than during the symbol-matching task in the alpha (9—12Hz), beta (13—40Hz) and low gamma (40—50Hz) bands across primarily central electrode sites. We observed no differentiation among difficulty conditions in either analysis.

To directly examine the potential group difference in the magnitude of SPD changes, we conducted pairwise contrasts on the brain scores from separate group analyses. We used an α value of 0.0042 to maintain family-wise error at 0.05 (0.05/12 = 0.0042). To examine whether there was a group difference in the magnitude of the change from resting-state to task, we contrasted resting-state to the average of the three difficulty conditions and calculated effect size. We found that for children, dynamic changes in SPD differentiated resting-state (*M* = 66.65, *SD* = 81.76) from the average of the three difficulty conditions (*M* = -22.22, *SD* = 2.18537), *t*(17) = 7.843, *p* < 0.001, *d* = 1.179, and for adults, dynamic changes in MSE differentiated resting-state (*M* = 52.66, *SD* = 119.3) from the average of the three difficulty conditions (*M* = 17.55, *SD* = 86.33), *t*(21) = 4.923, *p* < 0.001, *d* = 0.674. We contrasted each of the difficulty conditions to confirm a lack of differentiation among conditions of the symbol-matching task, and we did not find any significant differences amongst difficulty conditions for children or adults.

To ensure that the greater state-to-state modulations in SPD was not caused by the greater success of the difficulty manipulation for children, we used the same residualisation procedure as we used for MSE [38, 39] to remove the between-subjects variation related to response time (RT) and accuracy from the SPD data, and repeated our PLS analyses. In both cases, the lack of differentiation among difficulty conditions remained unchanged (RT, LV1, *p* < 0.001; accuracy, LV1, *p* < 0.001) and there was no change in the spatial extent of the effect. We then compared brain scores for resting-state and task conditions from the residualised analyses using t-tests and found significant differences only between resting-state and the average of the three difficulty conditions in each group. For brain scores residualised with RT in children, resting-state (*M* = 66.65, *SD =* 81.83) was higher than the average of all difficulty conditions (*M* = -22.22, *SD =* 68.19), *t*(17) = 7.845, *p* < 0.001, *d* = 1.180; in adults, resting-state (*M* = 52.66, *SD =* 119.26) was higher than the average of all difficulty conditions (*M* = -17.55, *SD =* 86.33), *t*(21) = 4.924, *p* = 0.001, *d* = 0.6745. For brain scores residualised with accuracy in children, resting-state (*M* = 66.65, *SD =* 81.76) was higher than the average of all difficulty conditions (*M* = -22.22, *SD =* 68.19), *t*(17) = 7.845, *p* < 0.001, *d* = 1.182; in adults, resting-state (*M* = 56.66, *SD =* 119.23) was higher than the average of all difficulty conditions (*M* = -17.55 *SD =* 86.32), *t*(21) = 4.924, *p* = 0.001, *d* = 0.6746.

**Fig 9:**
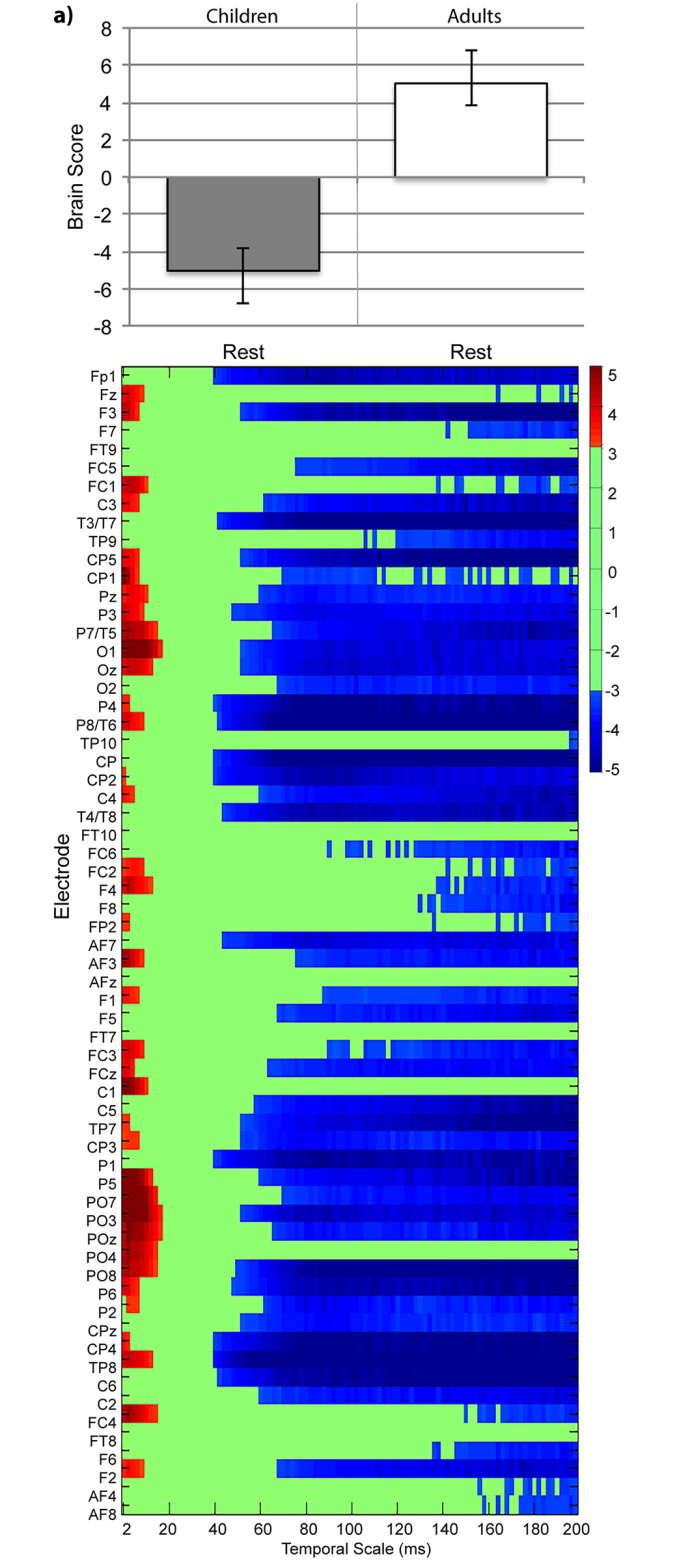
Task-PLS of MSE data from 10 second resting-state epochs in children and adults. The bar graph (top) shows averaged brain scores for effect of age group, with MSE. The statistical plots (bottom) show the effect of age group bootstrap ratios for each electrode and temporal scale. Positive bootstrap ratios (> 3.10) indicate regions that demonstrate the relationship between conditions as displayed in the bar graph (i.e., lesser MSE for children than adults). Negative bootstrap ratios (< -3.10) indicate regions that demonstrate the opposite relationship between conditions as displayed in the bar graph (i.e., greater MSE for children than adults).

SPD and MSE are related such that creating coarse temporal scales for MSE low-pass filters the data (29). Considering the main effect of group in the context of this relationship, we expect that our finding of SPD being higher for children than adults for the delta (0.5—3Hz) and theta (4—7Hz) bands would result in children having greater MSE for coarse temporal scales. However, the number of temporal scales included in the MSE analysis was limited to 30ms due to our use of a 1500ms epoch length. To further explore the spatiotemporal differences between children and adults, we created 10s epochs from resting-state data, allowing us to assess up to 200ms temporal scales. We used task-PLS to assess group differences and found that MSE during resting-state was higher in adults for temporal scales up to 25ms for electrode sites throughout the scalp. However, the relationship inverted for coarser temporal scales, so that children had greater MSE for temporal scales over 50ms at all electrode sites across the scalp (LV1, *p* < 0.001, Fig 9).

### Neuroimaging results comparing resting-state and within-trial epochs

#### MSE

**Fig 10:**
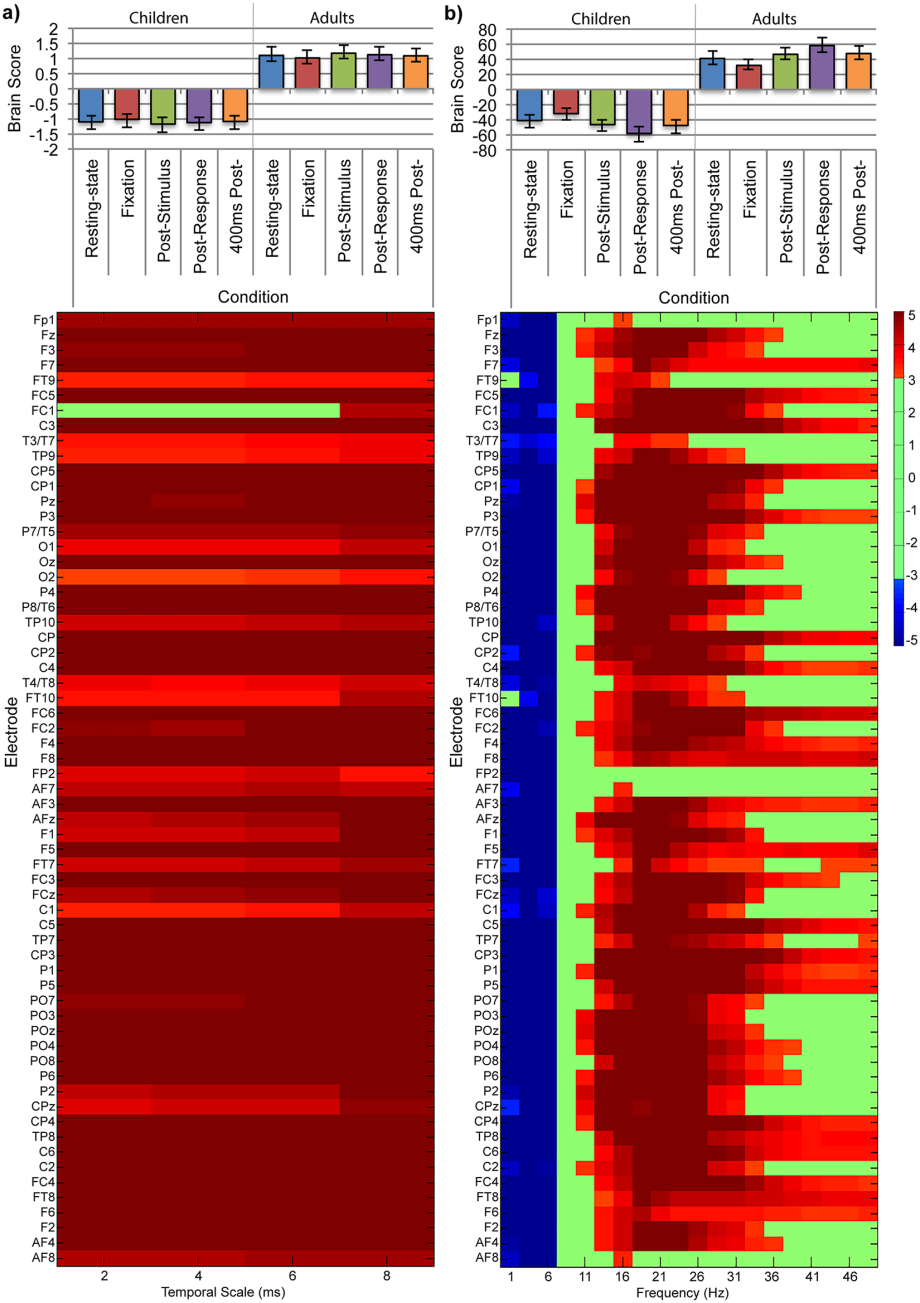
Nonrotated task-PLS of (a) MSE data and (b) SPD data for the resting-state and within-trial epochs analysis: LV1, main effect of age group (children and adults) with resting-state and within-trial epochs during symbol-matching conditions. The bar graph (top) shows averaged brain scores for the main effect of age group with (a) MSE and (b) SPD, with 95% confidence intervals derived from bootstrap estimation. The statistical plots (bottom) show the main effect of age group bootstrap ratios for each electrode and temporal scale. Positive bootstrap ratios (> 3.10) indicate regions that demonstrate the relationship between conditions as displayed in the bar graph (i.e., lesser (a) MSE and (b) SPD for children than adults). Negative bootstrap ratios (< -3.10) indicate regions that demonstrate the opposite relationship between conditions as displayed in the bar graph (i.e., greater (b) SPD for children than adults).

**Fig 11:**
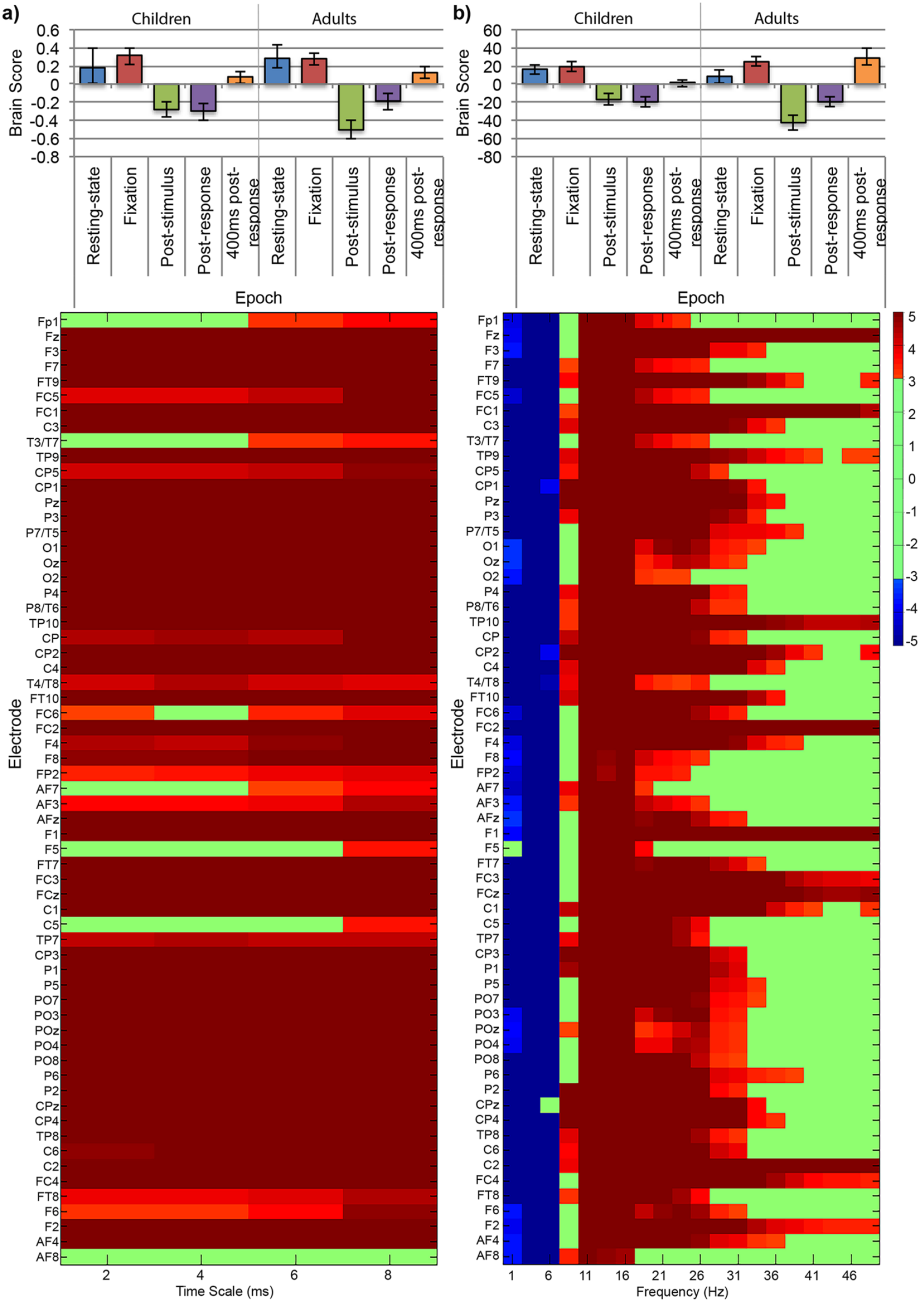
Nonrotated task-PLS of (a) MSE data and (b) SPD data for the two-group resting-state and within-trial epochs analysis: LV2, main effect of task (resting-state, fixation, post-stimulus, immediately post-response and 400ms post-response) in children and adults. The bar graph (top) shows averaged brain scores for the main effect of task with (a) MSE and (b) SPD, with 95% confidence intervals derived from bootstrap estimation. The statistical plots (bottom) show the main effect of age group bootstrap ratios for each electrode and temporal scale. Positive bootstrap ratios (> 3.10) indicate regions that demonstrate the relationship between conditions as displayed in the bar graph (i.e., greater (a) MSE and (b) SPD for resting-state, fixation, and 400ms post-response than for post-stimulus and immediately post-response). Negative bootstrap ratios (< -3.10) indicate regions that demonstrate the opposite relationship between conditions as displayed in the bar graph (i.e., lower (b) SPD for resting-state, fixation, and 400ms post-response than post-stimulus and immediately post-response).

We performed our within-trial analyses first with difficulty conditions separate and then averaged together. As both results were very similar (i.e., difficulty conditions were not differentiated for any epoch, and significance was similar across analyses), for the sake of parsimony we limit our report to the averaged results. We used nonrotated task-PLS of MSE scores to test for a main effect of age group (children vs. adults, contrast weights: 1, 1, 1, 1, 1, -1, -1, -1, -1, -1), a main effect of task/epochs within-trial (resting-state, fixation (pre-stimulus), and 400ms post-response vs. post-stimulus onset and immediately post-response, contrast weights: 2, -3, -3, 2, 2, 2, -3, -3, 2, 2), and an interaction between these effects (contrast weights: 2, -3, -3, 2, 2, -2, 3, 3, -2, -2). The main effect of age group was significant such that adults had greater brain signal variability than children (*p* < 0.001, Fig 10a). This effect was reliably expressed across all temporal scales tested (2 through 8ms) and across all electrode sites except FC1. The main effect of task/epochs within-trial was significant such that in both children and adults, brain signal variability was higher for resting-state, fixation and 400ms post-response, and lower for post-stimulus onset and immediately post-response (*p* < 0.001, Fig 11a). This effect was reliably expressed across all temporal scales across frontal, central, temporal, parietal, and occipital electrode sites. The interaction between group and task was not significant (*p =* 0.162). Thus, despite the lack of overlap in confidence intervals for post-stimulus onset between children and adults, the difference was not strong enough to produce a significant interaction. Therefore, we concluded that both the spatial extent and magnitude of MSE changes were similar for children and adults.

#### SPD

We used nonrotated task-PLS of SPD values to test for a main effect of age group (children vs. adults, contrast weights: 1, 1, 1, 1, 1, -1, -1, -1, -1, -1), a main effect of task/epochs within-trial (resting-state, fixation (pre-stimulus), and 400ms post-response vs. post-stimulus onset and immediately post-response, contrast weights: 2, -3, -3, 2, 2, 2, -3, -3, 2, 2), and an interaction between these effects (contrast weighs: 2, -3, -3, 2, 2, -2, 3, 3, -2, -2). The main effect of age group was significant (*p* < 0.001, Fig 10b), such that children reliably expressed greater SPD in the delta (0.5—3Hz) and theta (3—7Hz) bands across all electrode sites, and lesser SPD in high alpha (11—12Hz), beta (13—40Hz) and low gamma (40—50Hz) bands for all electrode sites except Fp2. The main effect of task/epochs within-trial was significant (*p* = 0.001, Fig 11b) such that resting-state and fixation were differentiated from post-stimulus onset and immediately post-response in children (the contribution from 400ms post-response was not reliable for children in this analysis). Resting-state, fixation and 400ms post-response were differentiated from post-stimulus and immediately post-response in adults. SPD differences were reliably lower in the delta (0.5—3Hz) and theta (3—7Hz) bands for resting-state, fixation, and 400ms post-response than post-stimulus onset and immediately post-response for all electrode sites. SPD was reliably greater in high alpha (11—12Hz) and low beta (13—15Hz) bands for resting-state, fixation than post-stimulus onset and immediately post-response in children, for all electrode sites, and in high beta (16—40Hz) and low gamma (40—50Hz) bands for many frontal, central, temporal, parietal and occipital electrode sites. The interaction between age groups and the task/epochs within-trial effect was not significant (*p* = 0.204). Again, despite the lack of overlap in some confidence intervals between children and adults, these differences did not produce a significant interaction. Therefore, we concluded that both the spatial extent and magnitude of MSE changes were similar for children and adults.

## Discussion

In the context of maturation from childhood to adulthood, we investigated state-to-state modulations in brain signal variability during resting-state and task, and examined how these changes relate to task performance. We found broad increases in brain signal variability during resting-state as compared to task, and showed that these increases were greater in spatial expression in children as compared to adults. We additionally found task difficulty-related modulations in children, where increased difficulty was associated with decreased variability. Thus, we demonstrated that MSE is an effective discriminator of internally- and externally-driven cognitive states in both children and adults.

### Children versus adults

With both our MSE analyses (comparing resting-state vs. the symbol-matching task, and resting-state vs. within-trial epochs), we replicated previous work suggesting that development yields an increase in MSE throughout the brain at fine and medium temporal scales (up to 28ms in previous literature [5, 6, 12]). Our analyses examined temporal scales up to 30ms, and at all temporal scales up to 24ms, we showed reliable increases in MSE throughout the brain, across all conditions and epochs. At temporal scales between 24 and 30ms, the effect weakened and became less widespread (i.e., the effect was constrained mainly to parietal electrodes). Our finding supports the idea that maturation is characterized by whole brain increases in local information processing (higher MSE at fine temporal scales) and mid-range interactions (higher MSE at middle temporal scales) with other neural populations [40, 41].

Our SPD results are complementary to our MSE results. As with MSE, we found a significant main effect of age group. For SPD, children had greater power in delta and theta bands, and adults had greater power in high alpha, beta and low gamma bands, with the effects being strongest up to 30Hz. These results are in line with previous literature that documents weaker contributions from lower frequencies (delta and theta) and greater contribution of higher frequencies (alpha, beta, and gamma) with increasing age [24, 42]. These changes also suggest that with increasing age, there is a relative increase in the contribution of local communication (supported by high frequencies) and a relative decrease in the contribution of long-range communication (supported by low frequencies) [43]. Upon first examination of our data, it might seem as if our SPD and MSE results are somewhat at odds with each other. Our combined resting-state and task MSE results show a monotonic increase from childhood to adulthood at temporal scales up to 30ms. The SPD results, on the other hand, showed that contributions from low frequencies decreased with age, but contributions from high frequencies increased with age. Constrained by the length of trials from our task data, we used an epoch length that allowed us to examine fine and medium, but not coarse temporal scales with MSE. Fine MSE temporal scales provide information about variability across all frequencies in the data, whereas coarser temporal scales are dominated by lower frequencies. Thus, we suspected that increased MSE at high and medium temporal scales reflected the age-related SPD increase at higher frequencies. The age-related MSE increase became weaker at medium temporal scales (24ms and above), as one would expect in the context of a greater contribution from the age-related SPD decrease at low frequencies. Based on this reasoning, we expected that MSE at coarse temporal scales would show an age-related decrease to reflect the age-related decrease in SPD at lower frequencies. To address this hypothesis, we tested MSE on resting-state data in children and adults. The resting-state (without task) analysis allowed us to examine temporal scales up to 200ms (using 10s epochs). We confirmed that children had lower MSE for fine and medium temporal scales (less than 24ms), but greater MSE for coarse temporal scales (greater than 45ms). Thus, unlike previous developmental studies which found monotonic MSE increases during maturation from childhood to adulthood (but examined only fine and medium temporal scales), our results suggest that maturation from childhood to adulthood is characterized by increased local information processing (higher MSE at fine temporal scales) and decreased long-range interactions with other neural populations (lower MSE at coarse temporal scales) [7, 40, 41]. This result is in line with MSE changes shown in the context of healthy aging throughout adulthood (as examples, see [7, 29, 41]). Overall, the comparison of our MSE and SPD results suggests that maturation is associated with changes in the linear dependencies that are evident in both MSE and SPD, rather than nonlinear dependencies that would be evident only in MSE [7, 26].

### Resting-state versus the symbol-matching task

We found that in both children and adults, brain signal variability was greater during resting-state than during task throughout the brain at all temporal scales that we examined, with the effect being strongest at central and parietal electrode sites. This is consistent with previous EEG work by Sleimen-Malkoun and colleagues [11], who also showed greater variability during resting-state at these temporal scales. In the context of our significant age group by task interaction, we additionally examined state-to-state modulations separately in children and adults. The resting-state to task MSE modulation was similar in effect size between children and adults, however, the spatial extent of this effect was much larger in children (i.e., throughout the brain in children versus localization primarily in central electrodes in adults). Previous research suggests that large-scale networks are more diffuse and less distinct in children as compared to adults [21, 22]. Therefore, it is possible that when children shift from internally-driven to externally-driven brain states, there are more regions involved, and therefore variability changes are more widespread.

In addition to the MSE modulation between resting-state and task, our analyses revealed a reliable difference in brain signal variability between high and low difficulty conditions for children but not for adults. This result reflects the fact that the difficulty manipulation was more effective in children than in adults. That is, children were generally slower and less accurate than adults, and differences in response time between high and low and between medium and low difficulty conditions were significantly greater in children than adults. Garret and colleagues [14] showed that brain signal variability decreases when task difficulty increases, because difficult conditions engage more cognitive resources, leaving lower capacity to prepare for other stimuli. Therefore, in our experiment, children needed to engage more resources to process the more difficult task. However, adults found all task conditions to be relatively easy, and did not show a difficulty-related modulation in brain signal variability. To confirm this interpretation, we residualised reaction time and accuracy from our MSE data to statistically remove the group differences in the effect of difficulty [38, 39]. In this context, the magnitude and spatial extent of the MSE differentiation between resting-state and task remained unchanged in both groups, but the differentiation among difficulty conditions for children disappeared. We concluded that when cognitive demands of state-to-state transitions are similar between groups, the MSE modulations are similar in magnitude but greater in spatial extent in children as compared to adults.

Again, our SPD results are complementary to our MSE results. As with MSE, we found a significant group effect, where SPD was different for resting-state versus task in children and adults. Resting-state was associated with decreased power in the delta and theta bands, and increased power in alpha, beta and low gamma bands. However, state-to-state modulations in SPD were more widespread for children than adults (i.e., throughout the scalp for children, and more posteriorly at fewer frequencies in adults), and also had greater magnitude. To our knowledge, no one has directly examined maturational effects on state-to-state changes in SPD, however, our SPD results are consistent with previous event-related potential (ERP) work. Compared to adults, the average evoked response in children tends to show higher amplitude and longer latency for early components, combined with less well-defined later components [6, 23–26]. Presumably, a larger amplitude response to an externally-driven state where ERPs are consistently produced compared to an internally-driven state where ERPs are not consistently produced could underlie the greater magnitude change we observed in SPD.

Interestingly, although children showed a performance-related MSE differentiation between high and low difficulty conditions, this differentiation was not evident in the SPD data. The SPD data, on the other hand, were more sensitive in the context of state-to-state changes where children showed a greater magnitude of change as compared to adults. This magnitude difference was not evident in our MSE data. These results suggest that performance-related modulations and state-to-state modulations have differing profiles of linear and nonlinear dependencies [7, 29].

### Resting-state versus within-trial epochs

We found that for children and adults, variability after stimulus onset and immediately post-response was lower than during fixation, 400ms post-response, and resting-state. The finding that variability levels are similar during fixation and resting-state suggests that in both cases, the brain in a ready state, from which available brain states may be efficiently explored [8]. These fine to middle temporal scale results, in combination with previous work showing that variability increases from resting-state to external task at coarse temporal scales [9, 11], suggest that it is specifically local information processing (higher MSE at fine temporal scales) and mid-range interactions with other neural populations (higher MSE at middle temporal scales) that supports the exploration of potential response options. Next, fine to middle-scale temporal scale variability decreases with stimulus onset, when the brain must settle into a response state to process and reliably transmit information [16]. Post-processing, brain signal variability returns to pre-stimulus levels. Cellular work in animals suggests that this increase in variability occurs only after task-related processing is complete [17, 18], when the brain is in a ready state for the next trial.

Interestingly, the lack of significant group by task interaction suggests that spatial extent of these transitions was similar between adults and children. This effect was widely distributed across all temporal scales tested (2 to 8ms) and in regions throughout the brain, and was so extensive in adults that the effect could not be even more extensive in children. Such widespread decreases in variability are consistently reported across animal and fMRI studies [17, 18, 44]. For example, Churchland and colleagues (44) found that firing rate variability declines with the onset of visual stimuli in all cortical regions tested in the macaque monkey and cat, with and without changes in mean firing rate. When measured with fMRI in human adults, He [15] found that variability reduced significantly in 86.8% of voxels, and only partially overlapped with regions that demonstrate mean signal change. It is important to note that the lack of group difference in spatial extent appears to contradict our resting-state versus symbol-matching task analysis results, in which we saw a more widespread effect in children than adults. However, it should be noted that the temporal scales used in these two analyses were quite different. Based on epoch length, we included temporal scales ranging from 2ms to 8ms in the resting-state versus within-trial epochs analysis, and from 2ms to 30ms in the resting-state versus symbol-matching task analysis. We show group differences in the spatial extent of the effect in the resting-state versus symbol-matching task analysis primarily for temporal scales greater than 15ms. These temporal scales were not included in the resting-state versus within-trial epochs analysis.

As with our previous analyses, the SPD results are complimentary to the MSE results. We found that SPD was similar among resting-state, fixation, and 400ms post-response epochs, and different from post-stimulus and immediately post-response in both children and adults. The effect on SPD was also very widespread, and was higher for resting-state, fixation, and 400ms post-response as compared to immediately post-response in the frequencies (i.e., high alpha, beta and low gamma bands) that dominate the MSE temporal scales examined (2 to 8ms). Thus, when considered with the MSE results, it appears as though both variability and SPD changes during the trial are similar in magnitude and quite widespread in both age groups.

### Limitations and conclusions

Certainly, there are limitations to our study. Our behavioural results indicate that children found the task more difficult than adults. Therefore, the identified group differences may be influenced by difficulty effects in addition to state-to-state change effects. Similarly, adults performed all of our difficulty conditions near ceiling. To try to tease out these difficulty effects from our data, we residualised response time and accuracy from our task data. Although we believe that our residualisation procedure adequately removed difficulty-related group differences, future studies should examine state-to-state variability changes in context where performance is matched for children and adults.

Despite these limitations, our results help elucidate maturational changes in state-to-state alterations between internally- and externally-driven states. Our study suggests that for children and adults, brain signal variability changes between resting-state and task had similar magnitude, but larger spatial distribution in children. Greater changes in task difficulty were associated with greater magnitude of modulation in MSE. However, this modulation was apparent only in children, as our difficulty manipulation had a greater effect in children. A comparison of our MSE and SPD results indicates that overall, these measures paint similar pictures of our data. However, maturational effects on resting-state to task transitions were stronger in SPD than in MSE analyses, whereas performance-related changes during task were stronger in MSE analyses. These results suggest that state-to-state modulations and performance-related modulations have differing profiles of linear and nonlinear dependencies in the data [29, 41]. Our results reinforce the idea that maturational brain changes manifest across different spatial and temporal scales affecting the information processing capacity, or complexity, of the brain.
